# PLD3 contributions to vascular dysfunction in the context of AD

**DOI:** 10.1002/alz70855_107171

**Published:** 2025-12-24

**Authors:** Elizabeth S Fisher, Kate Tubbesing, Katherine Stevens, Steven Lotz, James Hayes IV, Jessica Suiter, Thomas R Kiehl, Celeste M. Karch, Sally Temple, Taylor Bertucci

**Affiliations:** ^1^ Neural Stem Cell Institute, Albany, NY, USA; ^2^ Rensselaer Polytechnic Institute, Troy, NY, USA; ^3^ The Charles F. and Joanne Knight Alzheimer Disease Research Center, Washington University, St. Louis, MO, USA; ^4^ Department of Psychiatry, Washington University in St. Louis School of Medicine, St. Louis, MO, USA

## Abstract

**Background:**

Genome‐wide association studies (GWAS) have identified single‐nucleotide polymorphisms (SNPs) influencing the development and progression of Late‐Onset Alzheimer's Disease (LOAD), including low‐frequency variants. Phospholipase D3 (PLD3), an atypical phospholipase, harbors a rare variant, p.A442A, previously shown to double the risk of LOAD. PLD3 has been implicated in Amyloid Precursor Protein (APP) processing and Aβ development, potentially affecting amyloid deposition. Our research using bulk RNA sequencing (RNA‐seq) in mice suggested that PLD3 plays a role in vascular function; however, its specific role in endothelial cells (ECs) and supporting mural cells (pericytes, smooth muscle cells, and fibroblasts) remains unexplored.

**Methods:**

We investigated the impact of *PLD3* using APP/PS1xPLD3^KO^ mice and analyzing RNA‐seq from the cortex and comparing it to controls. Additionally, we utilized human‐induced pluripotent stem cells (iPSCs) to examine the effects of the *PLD3* variant and *PLD3* knockdown in ECs and mural cells. iPSCs were differentiated into ECs and mural cells and cultured separately in 2D or together in 3D vascular networks, which model EC‐mural cell interactions. We assessed *PLD3* variant and knockdown effects in purified ECs and mural cells through RNA‐seq, immunohistochemistry, and cytokine release. EC barrier function was evaluated using electric cell‐substrate impedance sensing (ECIS).

**Results:**

In APP/PS1xPLD3^KO^ mouse brains, pathways related to vascular function and inflammation were downregulated, while protein degradation pathways were upregulated. In iPSC models, *PLD3* variant and knockdown ECs exhibited altered cell division and APP processing pathways, with specific changes in AD‐GWAS genes *ADAM23, APOC1*, and *NRCAM*. *PLD3* variant mural cells showed disruptions in extracellular matrix remodeling, including *FN1*, and *MMP25*. In 3D vascular models, the *PLD3* variant led to altered IL‐8 release, which may have implications for vascular remodeling.

**Conclusion:**

Our findings demonstrate that *PLD3* influences vascular function and inflammation in mice, potentially contributing to disease pathogenesis. In iPSC‐derived vascular models, *PLD3* regulates EC proliferation and APP processing as well as mural cell matrix organization, suggesting that this variant may lead to vascular remodeling or instability. Future studies will explore the underlying mechanisms of these alterations and assess whether they can be targeted to prevent vascular dysfunction.

